# Atypical Manifestations of Old World Cutaneous Leishmaniasis: A Systematic Review and Clinical Atlas of Unusual Clinical and Specific Anatomical Presentations

**DOI:** 10.1002/hsr2.71273

**Published:** 2025-09-18

**Authors:** Bahareh Abtahi‐Naeini, Seyed Naser Emadi, Zabihollah Shahmoradi, Mahsa Pourmahdi‐Boroujeni, Ali Saffaei, Fereshte Rastegarnasab

**Affiliations:** ^1^ Pediatric Dermatology Division of Department of Pediatrics, Imam Hossein Children′s Hospital Isfahan University of Medical Sciences Isfahan Iran; ^2^ Skin Diseases and Leishmaniasis Research Center Isfahan University of Medical Sciences Isfahan Iran; ^3^ Skin Research Center of Razi and Imam Khomeini Hospital and Research Center for War‐affected People Tehran University of Medical Sciences Tehran Iran; ^4^ Student Research Committee Isfahan University of Medical Sciences Isfahan Iran; ^5^ Skull Base Research Center, Loghman Hakim Hospital Shahid Beheshti University of Medical Sciences Tehran Iran; ^6^ Department of Clinical Pharmacy, School of Pharmacy Shahid Beheshti University of Medical Sciences Tehran Iran; ^7^ Hypertension Research Center, Cardiovascular Research Institute Isfahan University of Medical Sciences Isfahan Iran

**Keywords:** atypical, clinical presentation, leishmania, old world cutaneous leishmaniasis

## Abstract

**Background and Aims:**

Cutaneous leishmaniasis (CL) represents the most common form of leishmaniasis. It imposes a significant medical burden due to long‐lasting ulcers and disfiguring scars, underscoring the need for comprehensive CL control strategies, particularly in endemic regions. This study aims to classify atypical CL presentations for clinical practitioners. Additionally, we compiled and categorized original images from our clinical encounters with CL to create a clinical atlas enhancing the existing literature.

**Methods:**

A systematic search for atypical manifestations of Old World CL was conducted in June 2023 via the PubMed database, utilizing MeSH‐based keywords including leishmaniasis, atypical, manifestation, and characteristics. All of the records were included. Exclusion criteria included records published before the 20th century, non‐English articles, review articles, nonclinical studies (experimental and epidemiological), studies focusing on New World CL (based on the study geographical location), or focusing on Post‐Kala‐Azar leishmaniasis. Relevant articles were selected and assessed.

**Results:**

Based on the clinical manifestations described in the selected studies, articles were classified into the following categories: (a) subacute CL, (b) chronic CL, (c) CL associated with lymphatic involvement, (d) CL associated with an immunocompromised state, and (e) cutaneous leishmaniasis on special anatomical sites. Clinical, diagnostic, and therapeutic facets were subsequently explored.

**Conclusion:**

The diagnosis of leishmaniasis can be challenging. Given the broadening spectrum of differential diagnoses for CL in clinical settings, dermatologists, pediatric dermatologists, internists, infectious disease specialists, and pediatricians must be aware of this classification of atypical CL.

## 1 Introduction

Cutaneous leishmaniasis (CL), the most prevalent form of leishmaniasis, is categorized into Old World (OWCL) and New World (NWCL) types based on geographical distribution [1]. The Old World is related to Asia, Africa, the Middle East, and southern Europe. While the New World is attributed to Central and South America [2].

In the Old World, *Leishmania* species such as *L. major, L. infantum, L. tropica, L. aethiopica*, and *L. donovani* are primarily responsible for the transmission of CL [1].

Transmission mechanisms of OWCL are either anthroponotic or zoonotic [3]. Zoonotic CL (ZCL) predominantly occurs in rural areas, is transmitted from animals to humans mainly by *L. major* and *L. infantum*, and typically resolves spontaneously within two to 4 months, although it may persist untreated for years. Conversely, anthroponotic CL (ACL) spreads from person to person mainly by *L. tropica* and *L. donovani*, predominantly in urban settings [4].

Beyond its substantial medical burden, CL is notorious for causing long‐term wounds and disfiguring scars, particularly in endemic regions, necessitating a comprehensive control strategy [5, 6]. Effective control in such areas requires the integration of prevention, early diagnosis, and effective treatment [7].

Diagnosing CL poses significant challenges. It may be misdiagnosed in non‐endemic areas and overdiagnosed in endemic ones [8, 9, 10]. Additionally, emerging diseases such as Coronavirus Disease 2019 (COVID‐19) and the potential coincidences with tuberculosis (TB) and human immunodeficiency virus (HIV), along with the increased prevalence of patients undergoing chemotherapy, can alter immune responses and induce states of immunocompromise [11, 12, 13]. These factors may lead to the presentation of atypical forms of CL, characterised by unusual clinical courses and patterns.

Atypical manifestations differ from the usual form mainly in terms of type, number, distribution, and location of the lesions or the clinical course of the disease. The classic form is usually defined as a papuloulcerative lesion, limited to an exposed area of the body, in a previously healthy patient, healing spontaneously [14, 15].

To date, no comprehensive systematic review consolidates findings on the atypical features of CL worldwide. Therefore, this review aims to compile data that will provide clinicians with a practical classification of atypical CL presentations. Additionally, we have compiled and categorized original clinical images from our experiences with CL to create a “clinical atlas”. This atlas aims to enhance the literature by providing a framework for classifying these presentations and offering diagnostic clues to assist clinicians.

## 2 Materials and Methods

### 2.1 Literature Search Strategy

This systematic review evaluates the atypical manifestations of Old World cutaneous leishmaniasis (OWCL). The review adhered to the Preferred Reporting Items for Systematic Reviews and Meta‐Analyses (PRISMA) guideline (Supplement 1). In collaboration with dermatology experts, pertinent keywords were identified from the MeSH databases for the literature search. The search was conducted in June 2023 using PubMed, focusing on MeSH‐based keywords including leishmaniasis, atypical, manifestation, and characteristics.

(Leishmaniasis[Title]) AND (Atypical[Title/abstract]), (Leishmaniasis[Title]) AND (Uncommon[Title/abstract]), (Leishmaniasis[Title]) AND (Unusual[Title/abstract]), (Leishmaniasis[Title]) AND (Not‐typical[Title/abstract]), and (Leishmaniasis[Title]) AND (Unconventional[Title/abstract]) were the searched queries.

The study is ethically approved by the “Research Ethics Committees of School of Medicine, Isfahan University of Medical Sciences, Isfahan, Iran” and the approval ID is: “IR.MUI.MED.REC.1402.376”. Written informed consent was obtained from the patients for the publication of identifying images or other personal or clinical details that compromise anonymity.

### 2.2 Inclusion and Exclusion Criteria

All of the records were included. Exclusion criteria included records published before the 20th century, non‐English articles, review articles, nonclinical studies (experimental and epidemiological), studies focusing on New World CL (based on the study geographical location), or focusing on Post‐Kala‐Azar leishmaniasis.

### 2.3 Study Selection and Appraisal

Records were retrieved and managed using EndNote X8 software (V8.0.1, Clarivate Analytics). The duplicated records were removed by the EndNote software function. The article selection process was divided into three phases to minimize selection bias. Initially, authors independently screened the titles, excluding irrelevant ones. Subsequently, the abstracts of the remaining articles were reviewed, and those deemed irrelevant were also excluded. Finally, efforts were made to obtain the full texts of the selected articles, including contacting corresponding authors as necessary.

Then, eligible studies were quality assessed using the Joanna Briggs Institute (JBI) Critical Appraisal Tools for case reports, case series, and cross‐sectional studies [16]. Two authors independently conducted this assessment, and any disagreements were resolved by a third author. The questions were answered using “Yes,” “No,” and “Not Applicable.”

### 2.4 Data Analysis

The remaining articles underwent a thorough evaluation, with comprehensive data extraction, including the country, year of study, population characteristics, sample size, atypical presentations, and other relevant parameters such as underlying disease, clinical course, lesion characteristics, location of the lesions, diagnostic methods, differential diagnosis, treatment regimens, and outcome. Then, the information extracted from the articles was organized and classified into atypical presentation categories based on our expert panel team.

Additionally, we compiled and categorized original clinical images from our encounters with CL to create a “clinical atlas”.

This atlas is intended to enhance the literature and assist clinicians in recognizing atypical forms of CL in their differential diagnoses.

## 3 Results

**Figure 1:**
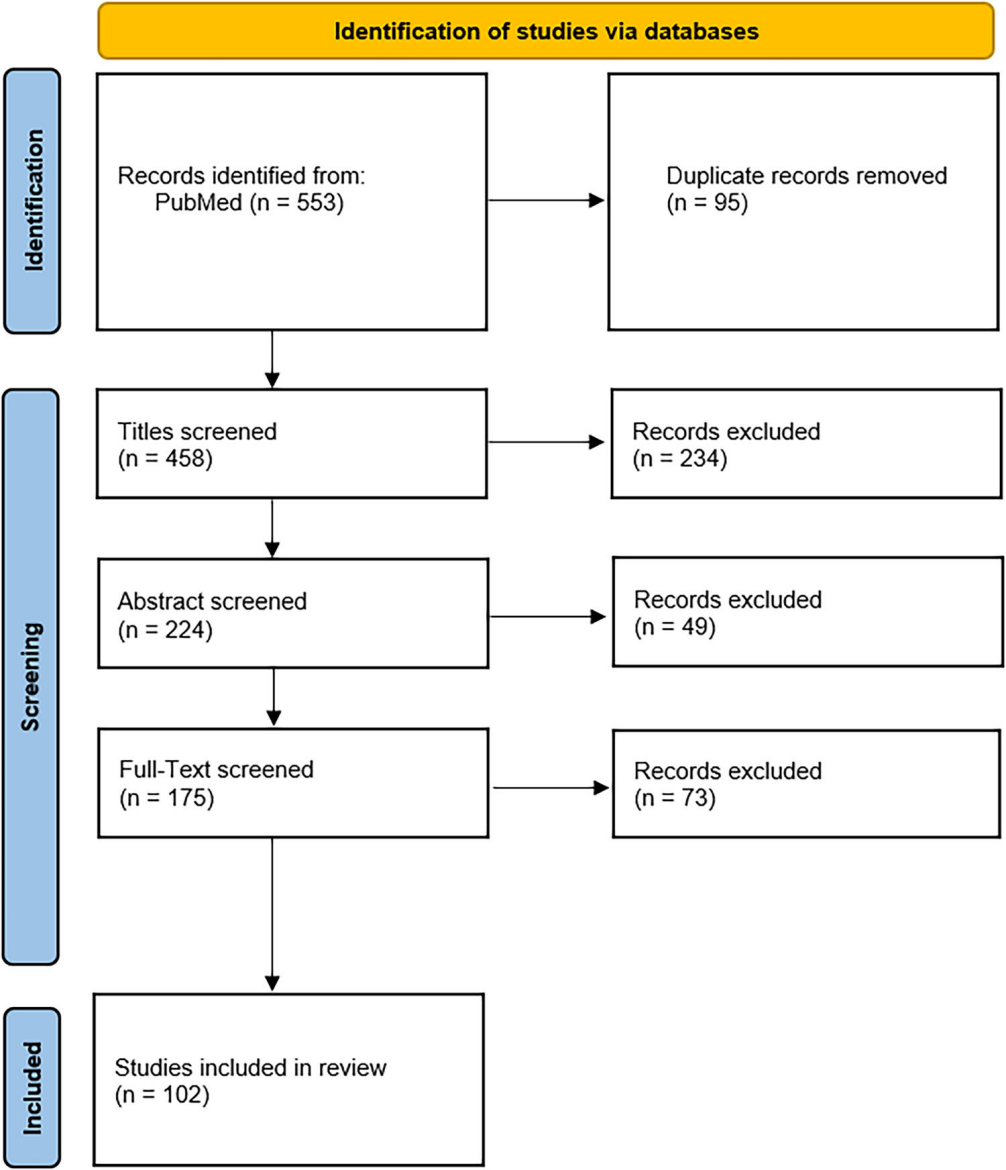
PRISMA‐flow diagram of the current review.

After executing the systematic search, we identified 533 articles, which were subsequently added to EndNote software. Removal of duplicate records by the EndNote software reduced the number to 458. A total of 283 articles were excluded following title and abstract screening. Upon full‐text screening, 175 articles were reviewed, and 102 were selected for inclusion. The selection process is illustrated in the PRISMA‐flow diagram depicted in Figure 1.

Supplement 2 represents the risk of bias assessment using JBI standard critical appraisal tools for the included studies.

Based on the content of the selected articles, atypical features of OWCL were categorized into the following groups:

- Subacute CL: Includes erysipelas‐like [17, 18, 19, 20, 21, 22, 23, 24, 25, 26], dermatomal [27, 28, 29], verrucous [30, 31, 32, 33, 34], tumor‐like [35, 36, 37, 38], eczematoid [39, 40], persistent ulcerative [41, 42], and infected CL [43].
- Chronic CL: Encompasses diffuse CL [44, 45, 46, 47, 48, 49, 50, 51], disseminated CL [52, 53, 54, 55, 56, 57], and leishmaniasis recidivans [58, 59, 60, 61, 62, 63, 64, 65, 66, 67, 68, 69, 70, 71, 72, 73, 74].
- CL Associated with Lymphatic Involvement: Features sporotrichoid CL [75, 76, 77, 78, 79, 80, 81] and localized Leishmania lymphadenitis [82, 83, 84].
- CL Associated with an Immunocompromised State: Coexistence with organ transplantation [85], HIV [86, 87, 88], diabetes [89], cancer chemotherapy, and biologic treatments [90, 91].
- Cutaneous leishmaniasis on Special Anatomical Sites: Includes acral [92, 93, 94, 95], oral and perioral [96], ocular and periocular [97, 98, 99, 100, 101, 102, 103, 104, 105, 106], nasal [107, 108, 109, 110, 111], auricular [112, 113, 114, 115, 116], and genitalia [117, 118].

Table 1 is a summary of the clinical characteristics and effective treatment options for each of the atypical features of OWCL.

**Table 1:** An overview of clinical Characteristics and treatment options of atypical features of old world cutaneous leishmaniasis.

Category	Characteristics	Treatment options	Treatment protocol	References
*Subacute*				
Erysipelas‐like	Erythematous, infiltrative, edematous, ulcerative, and crusted plaques Predominantly affecting the central face	IM meglumine antimoniate	10–60 mg/kg/d, 20–28 d	[17, 18, 19, 20, 21, 22, 23, 24, 25, 26, 119],
		IV sodium stibogluconate	20 mg/kg/d, 2–4 wks.	
		Oral miltefosine	150 mg/d, 28 d	
		Oral fluconazole	200 mg, 6 wks.	
		Systemic antibiotics:	15 mg/kg/d 1.5 g/d For 30 days	
		Clarithromycin Metronidazole		
		Cryotherapy	N/A	
Dermatomal	Satellite papules, nodules, and pseudo‐vesicular lesions arranged in a dermatomal pattern Mostly over‐exposed areas of the body	IM sodium stibogluconate	600 mg/d, 2 courses of 10 d	[27, 28, 29]
		Combination of:	20 mg/kg/d, 20 d 20 mg/kg/d, 30 d Every 2 wks.	
		IM meglumine antimoniate, Allopurinol, and Cryotherapy		
Verrucous	Non‐pruritic, painless verrucous plaques with a papillomatous and hyperkeratotic surface Typically found on the extremities, particularly the lower limbs	Combination of:	Weekly, 8 wks. N/A	[30, 31, 32, 33, 34, 72],
		IL meglumine antimoniate TCA 20%		
		IV sodium stibogluconate	20 mg/kg/d, 14–20 d	
		Combination of: 10% povidone‐iodine solution 2% ketoconazole cream	Once daily	
		Oral ketoconazole	400 mg/d, 8 wks.	
		Cryotherapy	Weekly, 4 months	
Tumor‐like	Ulcerative, verrucous, papulonodular, or nodular lesions Predominantly on the face, often affecting the nose and upper extremities	IM meglumine antimoniate	20 mg/kg/d, 28 d	[35, 36, 37, 38]
Eczematoid	Typically, lesions present on the extremities	IM meglumine antimoniate	20 mg/kg/d, 20 d	[39, 40]
Persistent ulcerative	Painful erythematous papules and pustules, ultimately ulcerate, revealing a yellowish exudate, pus discharge, or even a black crust with raised borders Lesions commonly occur on the upper and lower extremities	IM sodium stibogluconate	600 mg/d, 15 d	[41, 42, 120],
		IL sodium stibogluconate	600 mg/d, weekly for 3 wks.	
		IV amphotericin‐B	5 mg/kg/d, 14 d	
Infected	Single nodular lesion Involving the extremities or neck	NA	NA	[43]
*Chronic*				
Diffuse	Multiple discrete, papulonodular, non‐ ulcerated, keloid‐like, non‐tender lesions Involving the entire face, trunk, and extremities	Combination of:	20 mg/kg/d Every third day 1200 mg/d For 3 months	[44, 45, 46, 47, 48, 49, 50, 51]
		IV sodium stibogluconate IL sodium stibogluconate Oral pentoxiphyline		
		IM meglumine antimoniate	Two courses of 28 d	
		Ketoconazole	400 mg/d	
		Itraconazole	400 mg/d	
		Combination of:	600 mg/d 300 mg weekly	
		Rifampicin Levamisole		
Disseminated	Painless, papulonodular, ulcerated, crusted, and infiltrative lesions Affect the entire body, including palms and nails	Combination of:	20 mg/kg/d, 28 d Twice a day, 2 months Twice a day, 2 months	[52, 53, 54, 55, 56, 57]
		IM sodium stibogluconate Topical 25% zinc oxide cream 2% mupirocin ointment		
		Amphotericin‐B	3 mg/kg/d, 10 d	
		Combination of:	50 mg/d, 21 d 200 mg/d, 6 wks. Weekly, 6 wks.	
		Amphotericin‐B, Oral itraconazole, and Cryotherapy		
Recidivans	Erythematous, infiltrative, ulcerative, or crusted yellow‐reddish papules or granulomatous plaques Apple‐jelly‐like nodules Mostly involve the face	IL meglumine antimoniate	1 mL, twice a week for 3 wks. or weekly for 4 wks.	[58, 59, 60, 61, 62, 63, 64, 65, 66, 67, 68, 69, 70, 71, 72, 73, 74]
		IM meglumine antimoniate	15–20 mg/kg/d, 15–20 d	
		Systemic sodium stibogluconate	20 mg/kg/d, 14–28 d	
		Oral fluconazole	200 mg/d, 6 wks. (In children: 5 mg/kg/d, 4 wks.)	
		Oral ketoconazole	400 mg/d, 2 months	
		Combination of:	20 mg/kg/d, 20 d Weekly for 3 wks.	
		Systemic meglumine antimoniate Topical TCA 50%		
		Combination of:	60 mg/kg/d, 20 d 1 g/d N/A	
		Systemic meglumine antimoniate Ceftriaxone Topical Betamethasone and Gentamicin cream		
		Cryotherapy	N/A	
*Lymphatic involvement*				
Sporotrichoid	Painless erythematous papulonodular or even firm subcutaneous nodular lesions in a linear pattern Mostly on the upper extremities	IM meglumine antimoniate	20‐60 mg/kg/d, 14–21 d	[75, 76, 77, 78, 79, 80, 81]
		IL sodium stibogluconate	1‐2 mL (100 mg/mL), every 2 wks.	
		IV sodium stibogluconate	20 mg/kg/d, 14–20 d	
		Cryotherapy	N/A	
Lymphadenitis	Multilobulated, non‐tender, firm, and mobile erythematous nodules or papules	Systemic meglumine antimoniate	25 injections	[82, 83, 84]
		Oral miltefosine	100 mg/d, 4 wks.	
		Cryotherapy	N/A	
*Immunocompromised state*				
Organ Transplantation	Purple, painless, ulcerated nodule progressing to a painful, warty, and scabby lesion Lesions on the face or upper/lower limbs	Combination of:	300 mg/d 400 mg/d For 3 wks.	[85]
		Allopurinol Fluconazole		
HIV	Large crusted, ulcerative, maculopapular lesions or plaques Lesions all over the body, especially on the nose and upper extremities	Combination of:	60 mg/kg/d, 3 courses of 21 days N/A	[86, 87, 88]
		IM meglumine antimoniate Antiretroviral therapy (Lamivudine, Zidovudine, and Efavirenz associated with Cotrimoxazole)		
		Combination of:	N/A	
		Antiretroviral therapy (Lamivudine, Zidovudine, and Nevirapine) Co‐trimoxazole prophylaxis Oral fluconazole		
Diabetes	Giant multifocal ulcerated, erythematous plaques, or papulonodular crusted lesions Mainly on the lower limbs, the face, or the forearm	IM meglumine antimoniate	15 mg/kg/d, 20 d	[72, 89, 121],
		IV sodium stibogluconate	20 mg/kg/d, 14–20 d	
Posttreatment	Multiple large painless, erythematous, crusted ulcers Lesions on the upper limb	IM meglumine antimoniate	20 mg/kg/d, 21–28 d	[90, 91, 122],
		IV sodium stibogluconate	20 mg/kg/d, 2 wks.	
*Atypical anatomical location*				
Acral	A papule or furuncle‐like lesion progressing to a superficial ulceration with a serous exudate and crusting Might present as paronychia or dactylitis	IM meglumine antimoniate	20 mg/kg/d, 4 wks.	[92, 93, 94, 95]
		IL meglumine antimoniate	300 mg/mL for 0.5 mL at each session, 8 weks.	
		Combination of:	20 mg/kg/d, 20 d 2 months	
		IM meglumine antimoniate Topical sertaconazole		
		Surgical intervention	—	
Oral/Perioral	A small red raised lesion progressing into a large swollen purulent wound with bleeding and crust formation Might present as persistent perlesh or macrocheilia	IM meglumine antimoniate	20 mg/kg/d, 20 d	[92, 96, 123],
		IL meglumine antimoniate	300 mg/mL for 0.5 mL weekly, 8 wks.	
		Combination of:	Weekly 400 mg/d For 4 wks.	
		IL sodium stibogluconate Systemic ketoconazole		
Ocular/Periocular	A small papule that gradually enlarges as subcutaneous skin‐colored nodules, erythematous indurated plaques, chalazion‐like lesions, eczema‐like lesions, cancer‐like forms, or chronic granulomatous blepharitis Non‐tender, ulcerative lesions that might have a discharge, crust, and bleeding Might present as cellulitis or blepharitis	IM meglumine antimoniate	20 mg/kg/d, 20 d	[97, 98, 99, 100, 101, 102, 103, 104, 105, 106]
		20% paromomycin ointment	A choice for infants, for 2 months	
		IV liposomal amphotericin‐B	180 mg/d on days 1 through 5, 14, and 21	
		Surgical intervention	—	
Nasal	Localized eruption that enlarges as hyperkeratotic lesions, psoriasiform plaques, furunculous nodules, lupoid plaques, or rhinophyma‐like	IM meglumine antimoniate	20 mg/kg/d, 1 month	[107, 108, 109, 110, 111]
		IL meglumine antimoniate	N/A	
		Miltefosine	N/A	
		Surgical intervention	—	
Auricular	A small papule that gradually progresses to an enlarged, painful, erythematous, scaling, infiltrated nodule or plaque with prominent central ulceration May also present as zosteriform, erysipeloid, lupoid, sporotrichoid, eczematoid, hyperkeratotic, warty, or impetiginized lesions	IM meglumine antimoniate	20–60 mg/kg/d, 2 courses of 12–21 days interrupted by a 2‐week rest period	[112, 113, 114, 115, 116]
		IL meglumine antimoniate	Two injections separated by 15 days	
		IL Liposomal amphotericin‐B	2 mg/mL each session	
		Cryotherapy	N/A	
Genitalia	The glans and shaft of the penis are more likely to get involved. A small papule that gradually enlarges as a crusty, ulcerated nodule	IM meglumine antimoniate	20 mg/kg/d, 20 d	[117, 118]
		Oral miltefosine	150 mg/d, 28 d	

Abbreviations: d, day; IL, intralesional; IM, intramuscular; IV, intravenous; NA, not available; TCA, trichloroacetic acid; wks, weeks.

## 4 Discussion

In this systematic review, we aimed to investigate the clinical features of atypical Old World cutaneous leishmaniasis, clinical descriptions, diagnosis, and differential diagnosis. An effective therapeutic approach to these uncommon presentations may also be challenging. Also, original clinical images from our encounters with atypical presentations of CL are gathered as supplements.

## 5 Subacute Cutaneous Leishmaniasis

### 5.1 Erysipelas‐Like Cutaneous Leishmaniasis (Erysipeloid Type)

#### 5.1.1 Clinical Presentation

Erysipelas‐like CL, also known as erysipeloid type, typically begins as asymptomatic, painless papules that progress to erythematous, infiltrative, edematous, ulcerative, and crusted plaques predominantly affecting the central face, resembling erysipelas [20]. The lesions may exhibit either ill‐defined or sharply defined borders [17] (Supplement 3).

The rash may also extend to the forehead, nose, cheeks, and eyelids, forming a butterfly‐shaped pattern [18, 23]. Additionally, lesions can appear on extra‐facial areas, such as the upper or lower limbs [20, 22, 26]. This condition is predominantly observed in middle‐aged and elderly women [17, 18, 20, 21, 23, 24]. However, this condition should also be considered among young and male patients [124].

The skin quality alteration due to aging [21] and posttraumatic cutaneous lesions [21, 22] are the probable contributing factors to the development of erysipelas‐like CL [15].

#### 5.1.2 Diagnosis and Differential Diagnosis

Differential diagnoses for facial erysipelas‐like CL should include superficial skin infections, periorbital cellulitis, cold cellulitis, erysipelas, blastomycosis, leprosy, syphilis, tuberculosis, atypical mycobacterial infection, impetigo, acute lupus erythematosus, discoid lupus erythematosus, sarcoidosis, lupus vulgaris, and malignancies [17, 20, 24].

Parasitological studies remain the gold standard for diagnosing suspicious cases, involving Giemsa‐stained biopsy smears and detection of the Leishman body, histological examination, and polymerase chain reaction (PCR) [25].

Histopathological findings reveal dense mixed inflammatory cells and granulomatous infiltration with lymphohistiocytes, neutrophils, and plasma cells in the dermis with numerous intracellular amastigotes [20].

#### 5.1.3 Treatment

Treatment options for erysipelas‐like CL are varied, with the most commonly reported being pentavalent antimony compounds. Intramuscular (IM) injection of meglumine antimoniate (Glucantime) at doses of 10 mg/kg/day divided into two daily doses for 20 days [17] or 60 mg/kg/day [20] has been documented. Intravenous (IV) sodium stibogluconate at 20 mg/kg/day for 20 days is also reported but has been associated with hypokalemia and cardiac complications [25]. Intralesional (IL) administration of pentavalent antimonies is also reported as a therapeutic option, depending on the number, localization, and extension of the lesions. This increases drug concentration in the lesions, reduces systemic side effects, and costs [15].

Oral miltefosine (50 mg three times a day for 28 days) has shown positive effects for erysipelas‐like CL [23]. It has been used in cases with temporary benefits, but lesions relapsed after treatment cessation.

Oral fluconazole (200 mg daily for 6 weeks) has been employed as an alternative treatment with complete regression [22]. Systemic antibiotics such as clarithromycin [20] have also been reported as a successful therapeutic alternative. Another study reports the combination of 15 mg/kg/day clarithromycin and 1.5 g/day metronidazole for 30 days as a successful choice for a patient with left bundle branch block [119]. Cryotherapy is another option, particularly in older patients with underlying disease [20].

### 5.2 Dermatomal (Zosteriform) Cutaneous Leishmaniasis

#### 5.2.1 Clinical Presentation

Dermatomal CL, also known as zosteriform CL, is characterized by the appearance of satellite papules, nodules, and pseudovesicular lesions with or without an erythematous background arranged in a dermatomal pattern. This distribution provides a clinical hint towards diagnosing dermatomal CL [29]. Mostly, exposed areas of the body get involved, but there is also a report of involvement in covered regions such as the back and buttocks [27] (Supplement 4).

Cases of multi‐dermatomal CL have been reported, although the mechanism behind multi‐dermatomal involvement remains unclear; altered host immunity is suspected to play a role [27].

### 5.3 Diagnosis and Differential Diagnosis

The dermatomal distribution of lesions might initially suggest herpes zoster; thus, it should be considered in the differential diagnoses. A documented case of multi‐dermatomal CL was initially misdiagnosed and treated as herpes zoster [27].

Direct smear examinations help differentiate dermatomal CL from herpes zoster. A Tzanck smear from lesions shows numerous Leishman bodies [27]. Additionally, dermoscopy can aid in diagnosing dermatomal CL from other common differential diagnoses [28].

### 5.4 Treatment

Zosteriform CL generally responds to the standard therapy with meglumine antimoniate. Another treatment option is IM sodium stibogluconate at 600 mg/day for two courses of 10 days [29]. However, there is a report of multi‐dermatomal CL that resisted conventional therapy and was successfully treated with a combination of meglumine antimoniate, daily allopurinol, and cryotherapy every 2 weeks [27].

### 5.5 Verrucous Cutaneous Leishmaniasis (Warty CL)

#### 5.5.1 Clinical Presentation

Verrucous CL manifests as non‐pruritic, painless verrucous plaques with a papillomatous and hyperkeratotic surface, typically found on the extremities, particularly the lower limbs [32, 33]. Meanwhile, it is also seen on the upper limbs [30], nose, and lips [34] (Supplement 5).

#### 5.5.2 Diagnosis and Differential Diagnosis

The primary differential diagnosis for verrucous CL is the verrucous variant of squamous cell carcinoma [33]. Other considerations include warts, tuberculosis verrucosa cutis, verrucous carcinoma, and subcutaneous or deep mycosis [31, 32, 34].

Diagnosis typically begins with assessing epidemiological and clinical evidence and can be confirmed through tissue smears, skin biopsies with Giemsa staining, PCR, or histopathological examinations to identify leishman bodies [30, 33].

#### 5.5.3 Treatment

A combination of trichloroacetic acid (TCA) 20% with IL injections of meglumine antimoniate has been documented as a promising treatment option for verrucous CL [30]. IV sodium stibogluconate injection has also been reported as an effective option [72].

Additionally, atypical presentations of verrucous and pseudo‐tumoral CL have responded well to weekly cryotherapy sessions over 4 months [33]. Treatments using a 10% povidone‐iodine solution and 2% ketoconazole cream applied once daily have significantly improved lesions in some cases [34]. Furthermore, oral administration of 400 mg of ketoconazole daily for 8 weeks has led to complete resolution in another patient [32].

### 5.6 Tumor‐Like Cutaneous Leishmaniasis (Pseudo‐Tumoral CL)

#### 5.6.1 Clinical Presentation

Pseudo‐tumoral CL may manifest as flesh‐colored nodules distributed across the body [37]. This form typically presents with ulcerative, verrucous, papulonodular, or nodular lesions predominantly on the face, often affecting the nose and upper extremities. It exhibits a higher prevalence among pregnant women [31]. The lower extremities are less commonly affected [38] (Supplement 6).

#### 5.6.2 Diagnosis and Differential Diagnosis

Clinically, tumor‐like nodular CL may mimic various conditions such as cutaneous appendageal tumors, syringomas, trichoepitheliomas, lymphomas, pseudo‐lymphoma, lupus erythematosus, squamous cell carcinoma, and amelanotic melanomas, mainly when they manifest on the extremities [31, 35, 36].

While histopathological examination of tissue biopsies is instrumental in differentiating these lesions from actual tumors, PCR remains the most reliable method for detecting Leishmania‐specific DNA [37, 38].

Typical microscopic findings are mixed inflammatory infiltrates with many histiocytes and granuloma formation containing Leishman bodies [35].

#### 5.6.3 Treatment

IM meglumine antimoniate administered over 28 days has successfully healed pseudo‐tumoral CL lesions [37].

### 5.7 Eczematoid Cutaneous Leishmaniasis

#### 5.7.1 Clinical Presentation

Eczematoid CL typically presents with lesions primarily on the extremities, though they can also spread as diffused lesions over the trunk [40] (Supplement 7).

Eczematoid CL may be a result of epidermal invasion by *Leishmania*, followed by an intense cellular immune response, leading to significant inflammatory and eczematous changes [39].

#### 5.7.2 Diagnosis and Differential Diagnosis

Clinically, eczematoid CL can resemble eczema or acutely infected eczema, making it a consideration in persistent eczematous eruptions, especially in endemic regions [40].

Skin biopsy, histopathological examination stained with Wright‐Giemsa, and PCR can help to confirm the diagnosis [39].

#### 5.7.3 Treatment

The eczematous pattern of CL typically responds well to the standard treatment with pentavalent antimonials [40].

### 5.8 Persistent Ulcerative Cutaneous Leishmaniasis

#### 5.8.1 Clinical Presentation

Persistent ulcerative CL often presents as painful erythematous papules and pustules that gradually enlarge and ultimately ulcerate, revealing a yellowish exudate, pus discharge, or even a black crust with raised borders. These lesions commonly occur on the upper [42] and lower extremities [41] (Supplement 8).

#### 5.8.2 Diagnosis and Differential Diagnosis

The presentation of persistent ulcerative CL can resemble pyoderma gangrenosum, particularly in persistent lesions. Differential diagnoses should include pyoderma gangrenosum (PG), atypical bacterial, fungal, and mycobacterial infections, venous and arterial ulcers, and medium‐sized cutaneous vasculitis. Persistent ulcerative CL should be considered in the differential diagnosis of any nonresponsive cutaneous ulcer, especially in endemic areas [41, 120].

Microscopic examination of biopsies from these lesions often reveals numerous Leishmania parasites inside and outside the macrophages [41].

#### 5.8.3 Treatment

Persistent ulcerative lesions have shown successful regression through IL [42] or IM sodium stibogluconate (Pentostam) [41]. Another patient had an anaphylactic reaction associated with IM meglumine antimoniate. Therefore, the treatment had been switched to IV amphotericin‐B with promising results [120].

### 5.9 Infected Cutaneous Leishmaniasis (Impetiginized Cutaneous Leishmaniasis)

#### 5.9.1 Clinical Presentation

A study documented six patients with infected CL, each presenting a single nodular lesion on the extremities or neck [43] (Supplement 9).

#### 5.9.2 Diagnosis and Differential Diagnosis

Lesions may be misdiagnosed as suppurative folliculitis. Gram staining can help reveal leishmaniasis, especially within the central purulent areas [43].

## 6 Chronic Cutaneous Leishmaniasis

### 6.1 Diffuse Cutaneous Leishmaniasis

#### 6.1.1 Clinical Presentation

Diffuse cutaneous leishmaniasis (DCL) features multiple discrete, papulonodular, non‐ulcerated, keloid‐like, non‐tender lesions, varying from skin‐toned to erythematous [45]. These lesions may encompass the entire face [51], trunk, and extremities [45], with occasional involvement of palms and soles [46] (Supplement 10). Mucous membrane involvement is rare [44]. DCL may evolve from primary localized CL via direct extension, the bloodstream, or lymphatics [50].

An underlying immunogenetic condition and anergy due to a deficient cell‐mediated immune response are suspected contributing factors [46, 47].

#### 6.1.2 Diagnosis and Differential Diagnosis

Clinically, DCL may resemble lepromatous leprosy (LL), particularly regarding clinical appearance and distribution. Borderline tuberculoid leprosy [45], histoid Hansen′s disease, histoplasmosis, and PKDL should also be considered [46]. Unlike CL, LL features nerve involvement, which can be distinguished by a cutaneous sensation test [44]. PKDL can be excluded by the absence of visceral involvement, hepatosplenomegaly, lymphadenopathy, or other visceral signs of leishmaniasis [47].

Diagnostics include Giemsa‐stained skin smears and microscopic examination of skin biopsies [48].

#### 6.1.3 Treatment

DCL treatment responses are typically poor, with frequent relapses [46]. Reported treatments include IV and IL sodium stibogluconate at a dose of 20 mg/kg/day [51], IM meglumine antimoniate [45], and ketoconazole and itraconazole at 200 mg twice daily [44, 46, 49].

Combination of rifampicin at 600 mg daily and levamisole at 300 mg weekly has also been effective [47]. Combination therapy with cloxacillin, sodium stibogluconate, and paromomycin failed in one case; it was suggested to use amphotericin‐B as an alternative. Although, the patient refused and was discharged uncured [50].

DCL should be considered particularly in regions where both HIV and leishmaniasis are endemic, necessitating simultaneous treatment of both infections [48].

### 6.2 Disseminated Cutaneous Leishmaniasis

#### 6.2.1 Clinical Presentation

Disseminated cutaneous leishmaniasis typically starts with a primary lesion and spreads to cover extensive areas of the skin [57]. It is characterized by painless, papulonodular, ulcerated, crusted, and infiltrative lesions, which can affect the entire body, including palms and nails [54, 55, 56].

This form is reported in adults over 25 years of age with an immunosuppressive setting [52, 57], chronic obstructive pulmonary disease (COPD) [55], and vacuolar myelopathy [52].

#### 6.2.2 Diagnosis and Differential Diagnosis

Potential differential diagnoses include chronic PG, LL, lupus vulgaris, atypical mycobacterial infection, and subcutaneous or deep fungal infections [56, 57].

Diagnostic tools include skin biopsy, direct smear, and Giemsa staining, which help identify disseminated cutaneous leishmaniasis [54].

#### 6.2.3 Treatment

Antimonial agents are typically the first choice in the treatment of disseminated CL. Although these may improve symptoms [53], responses can be inconsistent due to the persistent nature of the condition, even after multiple treatment courses [54].

Combination of IM sodium stibogluconate injections and topical wound care with zinc‐oxide and mupirocin has shown successful results in a child involved with disseminated CL [53].

In two cases, glucantime was switched to amphotericin‐B due to the development of pancytopenia [52, 55]. Fluconazole was reported ineffective in another case [57]. Another patient was successfully treated with a combination of 50 mg of amphotericin‐B daily for 21 days, 200 mg of oral itraconazole, and weekly cryotherapy for 6 weeks [56].

### 6.3 Leishmaniasis Recidivans (Lupoid Leishmaniasis)

#### 6.3.1 Clinical Presentation

Leishmaniasis recidivans, also known as lupoid leishmaniasis, is a rare chronic form that typically follows an acute CL [58]. Although, there are two reports of cases with no obvious previous history of CL [64, 65]. Lupoid leishmaniasis is the most common atypical feature reported in a descriptive study in Pakistan [60].

After a variable period, the scar of a previously clinically healed CL is complicated by the peripheral development of erythematous, infiltrative, ulcerative [58], or crusted [61] yellow‐reddish papules or granulomatous plaques [62]. Apple‐jelly‐like nodules are the characteristic features of lupoid CL that can be seen on the periphery by diascopy [67, 69, 73]. A central atrophy may also be present [67, 73].

Lesions mostly involve the face [58, 70], but there are also a few reports about the involvement of the trunk and extremities [71]. Mucosal oral involvement may also be seen [61] (Supplement [Link], [Link]).

This type of CL may be due to insufficient treatment of previous CL, reactivation of dormant organisms following local trauma, surgery, topical steroids, or altered cellular immunity due to atopy [61, 63].

#### 6.3.2 Diagnosis and Differential Diagnosis

The primary differential diagnosis for lupoid leishmaniasis is lupus vulgaris due to their similar clinical and histological presentations, and both conditions typically feature a limited number of parasites [59, 69, 74]. Other differential diagnoses include cutaneous TB, rosacea granulomatous, lupus pernio [58], lupus erythematosus [69], discoid lupus erythematosus [61, 66], facial erysipelas [64], bacterial infections, lymphoma, pseudo‐lymphoma [62], verrucous carcinoma, squamous cell carcinoma [58], verruca plana [69], sarcoidosis, collagen vascular disease, and contact dermatitis [72]. Lesions on the lips may mimic syphilitic chancre [61], granulomatous cheilitis, Melkersson‐Rosenthal syndrome, and foreign body granuloma [62].

Diagnosis can be made through skin biopsy, smear, Giemsa staining, histological examination, PCR, and fine‐needle aspiration cytology (FNAC) from lymph nodes. Histopathological findings reveal a granulomatous reaction with various inflammatory cell infiltrations [67, 70, 71, 73].

#### 6.3.3 Treatment

Lupoid leishmaniasis is mainly treated by IM [62] or IL [67, 69] meglumine antimoniate injections. This may be added to systemic antibiotics such as ceftriaxone and topical care [58]. In this case, systemic meglumine antimoniate injections caused a prolonged corrected QT interval (QTc) for a day [58].

When treatment with doxycycline at 200 mg/day was insufficient, oral fluconazole at 200 mg/day successfully healed the lesions [65]. Oral fluconazole 5 mg/kg/day in a child [62] and oral ketoconazole at 400 mg daily [64] have been effective.

Daily IM [70] and IV [72] sodium stibogluconate injections are also effective treatments. A recalcitrant case showed marked improvement after treatment with a combination of topical TCA and systemic glucantime [68]. Cryotherapy has also been used with promising clinical benefit [62, 73].

## 7 Cutaneous Leishmaniasis Associated With Lymphatic Involvement

### 7.1 Sporotrichoid Cutaneous Leishmaniasis

#### 7.1.1 Clinical Presentation

Sporotrichoid cutaneous leishmaniasis is a rare feature of CL, with lymphatics spreading from the primary lesion and the inflammation of lymph nodes among dermal and subcutaneous lymphatics [81].

The disease presents as painless erythematous papulonodular or even firm subcutaneous nodular lesions in a linear pattern, mostly on the upper extremities [75] (Supplement 13).

Some reports suggest that procedures performed on the initial lesion (e.g., biopsy, tissue‐damaging treatments [75], or intralesional injections [81]) may contribute to lymphatic dissemination by stimulating local inflammation or disrupting tissue integrity. However, not all cases of lymphatic spread were associated with lesion manipulation in our review. This implies that other contributing factors, such as host immune response and parasite virulence, may also play a role.

In a case, ulcerated lesions presenting as Leishmanial whitlow, Leishmanial paronychia, and sporotrichoid spread have also been reported [78]. Recurrent CL presenting as sporotrichoid abscesses is also reported [76, 79].

#### 7.1.2 Diagnosis and Differential Diagnosis

This presentation resembles the typical form of sporotrichosis [78]. Atypical mycobacteria, Nocardia, and squamous cell carcinoma should be considered as other differential diagnoses [72, 75]. Skin biopsy, smear, Giemsa stain, and PCR can be used to diagnose [81]. Histopathological investigations in a patient revealed panniculitis [77].

#### 7.1.3 Treatment

Sporotrichoid CL should be treated with systemic anti‐leishmanial agents. Meglumine antimoniate IM injection is the first choice [77, 78, 79]. Although, this treatment caused pericarditis in a case [77]. IV and IL sodium stibogluconate are also reported to be successful [72, 80, 81]. Cryotherapy can also be helpful [78].

### 7.2 Localized Leishmania Lymphadenitis

#### 7.2.1 Clinical Presentation

Localized Leishmania lymphadenitis is a benign transient phenomenon with inflammatory changes within isolated lymph nodes without systemic involvement [83]. It may be caused by the lymphatic dissemination of macrophages transporting amastigotes, or the migration of Leishmanial antigen by Langerhans cells from skin to lymph nodes [83].

Localized Leishmania lymphadenitis can be multilobulated, non‐tender, firm, and mobile erythematous nodules or papules. Lymph nodes are enlarged and may present as masses [82, 83]. Enlarged lymph nodes may be without cutaneous involvement [82, 84].

#### 7.2.2 Diagnosis and Differential Diagnosis

Lymph node involvement in this disease may be misdiagnosed as a complication of secondary bacterial infection [83], tuberculosis, toxoplasmosis, or cat‐scratch disease [82].

Sporotrichoid cutaneous leishmaniasis is characterized by linear arrangements of nodular or papular lesions extending along dermal or subcutaneous lymphatic pathways from a primary site [75]. In contrast, localized Leishmania lymphadenitis presents with isolated regional lymph node enlargement, maybe without a visible dermal component [82, 83].

Microscopic evaluation of resected masses following cervical and parotid excision confirmed the diagnosis in a patient [83]. Skin‐stained smears [82], lymph node biopsy, fine needle aspiration, and cytology smears [84] are the reported ways to diagnose.

#### 7.2.3 Treatment

Localized leishmania lymphadenitis should be treated systemically. There is a report of a patient treated with 25 injections of meglumine antimoniate [83]. Another patient was treated with oral miltefosine 50 mg twice daily for 4 weeks [84]. Cryotherapy is also reported as a helpful method to heal the lesions [83].

## 8 Cutaneous Leishmaniasis Associated With an Immunocompromised State

### 8.1 Coexistence With Organ Transplantation

#### 8.1.1 Clinical Presentation

CL has been reported in association with solid organ transplantation, particularly renal transplants. However, the frequency and types of solid organ transplants in populations exposed to Leishmania species were not specifically analyzed in this review [85].

It may present as a purple, painless, ulcerated nodule progressing to a painful, warty, and scabby lesion. Lesions may be on the face or upper/lower limbs [85].

#### 8.1.2 Diagnosis and Differential Diagnosis

This presentation may be misdiagnosed as an atypical cutaneous mycobacteriosis, bacillary angiomatosis, pyogenic abscess, squamous cell carcinoma, or cutaneous Kaposi sarcoma [85]. CL should be carefully considered in immunosuppressed organ transplant recipients, especially in endemic areas.

It can be diagnosed with skin smear, histopathological examination, or PCR [85].

#### 8.1.3 Treatment

Standard pentavalent antimonial therapy in patients with organ transplantation may cause adverse effects such as pancreatitis and nephrotoxicity [85]. Intradermal meglumine antimoniate showed no clinical improvement in a kidney transplant recipient, and the lesion worsened to a pseudo‐tumoral appearance. Although, the patient was successfully treated with a combination of allopurinol and fluconazole for 3 weeks [85].

### 8.2 Coexistence With HIV

#### 8.2.1 Clinical Presentation

Leishmaniasis may be the first presentation of underlying HIV infection. Especially in patients with concomitant weight loss and leishmaniasis, HIV co‐infection should be considered [88]. It may also be the result of previous leishmaniasis reactivation due to the HIV‐related decreased host immunity, mostly with CD4^+^ count less than 100 [88]. HIV‐coinfected patients with leishmaniasis usually show a variety of multiple disseminated and atypical cutaneous lesions [88].

It initially may present as erythematous lesions that progress to large crusted, ulcerative, maculopapular lesions [88] or plaques [86]. Lesions may occur all over the body, especially on the nose and upper extremities [88]. It may lead to an atypical mucocutaneous, obstructive nasal mass [86]. Recurrent similar lesions at the same previous site are probable [88].

#### 8.2.2 Diagnosis and Differential Diagnosis

Granuloma, malignancy, and disseminated TB are reported as differential diagnoses [88]. Skin biopsy and smear are standard diagnostic methods in these patients [86].

#### 8.2.3 Treatment

Although there is a case series of patients treated successfully with meglumine antimoniate and antiretroviral therapy in HIV patients [88], several studies report poor response, potential toxicity, and frequent relapses in HIV‐infected patients who underwent treatment with standard anti‐leishmanial regimens [86, 87]. A combination of antiretroviral therapy, co‐trimoxazole prophylaxis, and oral fluconazole is reported as a successful therapeutic option [86].

### 8.3 Coexistence With Diabetes Mellitus

#### 8.3.1 Clinical Presentation

Defective cellular immunity due to diabetes mellitus (DM) may lead to a weak response against Leishmania parasites and cause an atypical clinical feature [89].

It may present as giant multifocal ulcerated, erythematous plaques, or papulonodular crusted lesions mainly on the lower limbs, the face, or the forearm [89] (Supplement 14).

#### 8.3.2 Diagnosis and Differential Diagnosis

PG, lymphoma, atypical mycobacterium, and deep fungal infection are reported as other differential diagnoses [72].

This feature can be diagnosed with a skin biopsy, Giemsa‐staining skin smear, PCR, or a histopathological examination [89].

#### 8.3.3 Treatment

In patients with diabetes mellitus, lesions may improve with systemic meglumine antimoniate for 20 days [89] or systemic sodium stibogluconate [72, 121].

### 8.4 Coexistence With Cancer Chemotherapy or Immunosuppressive Treatment

#### 8.4.1 Clinical Presentation

Immunosuppressive medications can alter the course of CL. These patients can experience more severe symptoms [90, 91]. Multiple large, painless, erythematous, crusted ulcers on the upper limb are reported in patients with leukemia [91]. Also, reactivation and dissemination of CL in a rheumatoid arthritis patient under treatment with systemic corticosteroid is reported. The patient presented with extensive reddish‐purple papules and inflammatory, erythematous, ulcerated, hyperkeratotic, crusted plaques on the face, scalp, torso, and limbs [90] (Supplement 15).

#### 8.4.2 Diagnosis and Differential Diagnosis

Herpes simplex virus (HSV), psoriasis, sarcoidosis, sweet syndrome, mycobacterial infection, and connective tissue diseases are listed as differential diagnoses for these lesions [90].

Diagnostic methods include skin biopsy [91], Giemsa‐staining skin smear, histopathology, and PCR [90].

#### 8.4.3 Treatment

IM injections of meglumine antimoniate and IV sodium stibogluconate improved the lesions of immunocompromised patients under cancer chemotherapy or immunosuppressive treatments [90, 91, 122].

## 9 Cutaneous Leishmaniasis on Special Anatomical Sites

### 9.1 Acral CL: Dactylitis, Leishmaniasis Paronychia

#### 9.1.1 Clinical Presentation

Fingers are not a common site for CL (0.3%) but can be involved as paronychia or dactylitis. Initially, it presents as a papule or furuncle‐like lesion and eventually progresses to superficial ulceration within 2–3 weeks. The ulcer usually has a serous exudate and crusting [94, 95].

In Paronychia, nail folds are erythematous, swollen, and tender with no pruritus (Supplement 16).

In nail plate involvement, it may exhibit longitudinal depression of the nail bed to destruction of the nail apparatus [93, 94]. In dactylitis, sausage digit deformity may present [95]. It may also present a painless fissure‐like lesion in a horizontal anatomical fissure without discharging [92].

#### 9.1.2 Diagnosis and Differential Diagnosis

Differential diagnoses should be considered, including bacterial or fungal infections, fistulated mucoid cysts, Bowen disease, psoriasis, and malignancies such as epidermoid carcinoma [93, 95].

The diagnosis is typically made through histological examination of a slit‐skin smear stained with Leishman or Giemsa stain. Also, biopsy and PCR can be helpful. Moreover, FNAC for cytological examination can be used for fissure‐like cases [92, 94, 95].

#### 9.1.3 Treatment

The treatment of choice for acral CL is pentavalent antimonial compounds either directly or systemically. However, treatment should be individualized. As the lesion is small, direct injection seems to be preferred. In significantly expanded inflammatory lesions, systemic therapy for 3–4 weeks might be a reasonable option [94]. IL injections of meglumine antimoniate, 300 mg/mL for 0.5 mL at each session for 8 weeks, are reportedly effective for fissure‐like CL in fingers [92].

IM injection of meglumine antimoniate, in addition to topical sertaconazole, is another effective option [95]. Surgical intervention, especially for leishmaniasis paronychia, can bring better clinical outcomes [93].

### 9.2 Oral and Perioral Leishmaniasis: Labial CL, Perlesh, and Cheilitis

#### 9.2.1 Clinical Presentation

CL on the lips can be presented as a small red raised lesion that progresses into swelling and even large wound formation. The lesion may be painful or painless, accompanied by purulent discharge, bleeding, and crust formation. Regional lymphadenopathy may also be present. The lesion may extend into the labial mucosa, causing a significant induration (Supplement 17A).

Rarely, CL on the lip might gradually form a fissure‐like lesion in oral commissures and present as a persistent perlesh [92, 96] (Supplement 17B). Macrocheilia, the swelling of one or both lips with a central crusted ulceration, is also attributed to CL as one of the primary causes [123].

#### 9.2.2 Diagnosis and Differential Diagnosis

There is a report of a 13‐year‐old boy with a devastating disfigurement after getting misdiagnosed as having herpes and a bacterial infection [36]. A fissure‐like presentation of CL on the lip region can be mistaken for a staphylococci infection. Other forms should also be differentiated from insect bite reaction or cutaneous TB [92, 96].

Diagnosing CL on the lip can be challenging since Leishmania parasites are usually absent in slit‐skin smears. A skin biopsy from the edge of the lesion may show a well‐defined lymphocyte granuloma, but it is not diagnostic. FNAC is a better choice as it can demonstrate Leishmania trophozoite bodies with a sensitivity of 89% and specificity of 100%. In lymphadenopathy, FNAC of lymph nodes can also be diagnostic [92, 96]. PCR may also be a useful diagnostic option [123].

#### 9.2.3 Treatment

Systemic meglumine antimoniate remains the standard treatment [123]. However, it can be administered intralesional at a dose of 300 mg/mL for 0.5 mL weekly for 2 months in patients involved with oral and perioral CL [92]. There is another report of successful treatment using IL sodium stibogluconate once a week plus systemic ketoconazole 400 mg daily for 4 weeks [96].

### 9.3 Ocular and Periocular Cutaneous Leishmaniasis: Cellulitis and Blepharitis

#### 9.3.1 Clinical Presentation

Eyelid involvement is uncommon and accounts for only 2%–5% of cases with facial involvement. The frequent movements of the eyelid make it a less likely site for biting [98].

CL on the eyelid typically begins with a small papule that gradually enlarges over time. There are various clinical presentations of eyelid CL, including subcutaneous skin‐colored nodules, erythematous indurated plaques, chalazion‐like lesions, eczema‐like lesions, cancer‐like forms, and chronic granulomatous blepharitis. The lesion is usually non‐tender, ulcerated, and might have a discharge, crust, and bleeding [97, 98, 100, 101, 102, 106]. It can also present as a huge tumor‐like mass on the eyelid, leading to severe mechanical ptosis [97] (Supplement 18).

Rarely, eyelid CL can progress to pre‐septal cellulitis, which presents as hyperemia and edema of both eyelids and spreads to other areas of the face with pre‐auricular lymphadenopathy [105].

Chronic eyelid CL can spread to conjunctiva, sclera, and cornea. Patients may inadvertently inoculate their conjunctiva with the parasite by their finger, causing destruction of various ocular tissues and presenting as trichiasis, eyelash loss, dacryocystitis, ptosis, lagophthalmos, entropion, ectropion, corneal opacity, scleromalacia, scleral perforation, intraretinal hemorrhage, optic neuropathy, and blindness [100, 103]. Pediatrics may be more susceptible to these complications [102].

Ocular structures can also become infected through the hematogenous route and develop several rare complications such as cataracts, binocular uveitis, narrowing of the anterior chamber, decreases in visual acuity, granulomatous formation and vascularization on the iris, and scleral perforation [103].

#### 9.3.2 Diagnosis and Differential Diagnosis

The differential diagnosis for eyelid CL includes secondarily infected insect bites, furuncles, hordeola, chalazion, eyelid eczema, histoplasmosis, sporotrichosis, rhinoscleroma, dacryocystitis, blepharitis, TB, syphilis, impetigo, sarcoidosis, and malignancies such as basal cell carcinoma, squamous cell carcinoma, and keratoacanthoma [98, 100, 101, 102]. Sebaceous carcinoma should be considered an essential differential diagnosis in chronic relapse of nodular lesions [101, 102].

Diagnosis of eyelid CL can be challenging due to the potential difficulty of sampling and bacterial growth in the lesion culture [98, 100]. A direct smear from the edge of lesions might be enough to diagnose. Nevertheless, a direct slit‐skin smear of eyelid eruption may help [97], but it is not sensitive enough. PCR and electron microscopy studies are much more convenient [99, 102, 106].

#### 9.3.3 Treatment

Since the eyelid is an important anatomic site both cosmetically and functionally, it requires systemic treatment to ensure a better outcome. Generally, IM meglumine antimoniate at 20 mg/kg for 20 days has shown definite improvement with no relapses [97, 98, 102].

In one report, a paromomycin ointment of 20% was used for eyelid CL in an infant, which resulted in a complete resolution with no relapses [102]. If the patient cannot tolerate the meglumine antimoniate, which can be particularly problematic in elderly patients, treatment can be replaced with systemic liposomal amphotericin‐B (180 mg/day IV on days 1 through 5, 14, and 21) [105].

In cases of complicated ocular involvement, specialized ophthalmology services such as surgical intervention may be required. However, ocular CL can usually be associated with permanent and incurable scars and impairment of visual acuity [103, 104]. Therefore, early diagnosis and intensive treatment may prevent ocular complications, especially in individuals with a previous history of leishmaniasis [103, 106].

### 9.4 Nasal Cutaneous Leishmaniasis and Cyrano Nose

#### 9.4.1 Clinical Presentation

Nasal involvement in CL is observed in 9.82% of cases. Due to being an immobile projected part of the face, the nose is more susceptible to being bitten. Initially, it appears as a localized eruption that enlarges over time [111]. It can cause severe deformities like septal mutilation [109]. Various forms of nasal involvement have been reported, such as hyperkeratotic lesions, psoriasiform plaques, furunculous nodules, lupoid plaques, and rarely rhinophyma‐like (Supplement 19). Rhinophyma‐like CL presents as a soft, painless nose swelling clinically resembling rhinophyma [107, 108, 111].

#### 9.4.2 Diagnosis and Differential Diagnosis

Differential diagnoses include granuloma faciale, lupus pernio, impetigo contagiosa, sarcoidosis, sebaceous adenoma and carcinoma, squamous cell carcinoma, basal cell carcinoma, angiosarcoma, cutaneous B‐cell lymphoma, mycobacterium infection, Cryptococcus, and rhinoscleroma [107, 108, 110].

Fine needle sampling can be diagnostic. Microscopic studies might yield false negative results, whereas PCR is a sensitive assessment tool that can also determine the responsible species.

#### 9.4.3 Treatment

As the nose is a sensitive anatomic and cosmetic location, systemic treatment of CL in this unit is preferred. However, IL antimonial therapy is also reported to be successful [111]. Systemic miltefosine and systemic antimony salt can be appropriate therapeutic choices. Any delay in the initiation of the treatment can be associated with more complications that may require surgical interventions [107, 108].

### 9.5 Auricular Cutaneous Leishmaniasis and Chondrodermatitis

#### 9.5.1 Clinical Presentation

An auricular CL lesion is a painful, erythematous, scaling, ulcerated lesion on any part of the auricle. This condition is characterized by the onset of a small papule that gradually progresses to an enlarged infiltrated nodule or plaque with prominent central ulceration (Supplement 20). Ulcers may ooze, bleed, and develop seropurulent crusting. Induration may also expand to the parotid region, while auditory thresholds commonly remain unchanged. Regional lymphadenopathy may also be present [112, 115].

Auricular CL may also present as zosteriform, erysipeloid, lupoid, sporotrichoid, eczematoid, hyperkeratotic, warty, and impetiginized lesions [115].

#### 9.5.2 Diagnosis and Differential Diagnosis

Auricular CL can be listed with other differential diagnoses, including relapsing polychondritis, leprosy, atypical mycobacterial infection, deep fungal infections, syphilis, Winkler disease, bites, sarcoidosis, granulomas, neoplasms such as squamous cell carcinoma, lupus vulgaris, discoid lupus, and lymphoma [112, 114, 115].

Patients often have a history of various prescribed drugs with partial response of signs and symptoms. They may also have undergone surgical debridement with suspicion of perichondritis. Usually, laboratory testing and imaging in immunocompetent patients have average normal results [113, 114, 116].

Detailed history taking with attention to geographical prevalence may facilitate diagnosis. Similar lesions on other sides can be a clue. Usually, diagnosis is based on a direct smear, biopsy, or PCR. Nevertheless, a biopsy or smear might be false negative. It may be interpreted as squamous cell carcinoma [112, 114, 115].

#### 9.5.3 Treatment

Auricular CL treatment protocol should be individualized, but mainly has a component against the parasite, including meglumine antimoniate and liposomal amphotericin‐B [112, 113, 114]. Meglumine antimoniate can be administered directly to the lesion site twice at a 2‐week interval or systemically. Even though the IL injection brought a complete resolution, the preferred treatment in most cases of ear involvement is systemic administration, but with a higher risk of chronicity and undesirable cosmetic outcomes [112, 114].

Nevertheless, multiple episodes of IM and IL sodium stibogluconate in addition to episodes of cryotherapy were unsuccessful for a patient in Sri Lanka [26].

In a pediatric case with auricular CL, IV liposomal amphotericin‐B accompanied by topical paromomycin showed unwilling results. However, 2 mg/mL IL liposomal amphotericin‐B injection was associated with a complete response [113]. Cryotherapy can also be used as an adjuvant therapy [112].

### 9.6 Genitalia Leishmaniasis

#### 9.6.1 Clinical Presentation

Generally, CL in the genital area is rare due to being covered with clothing. A common clinical scenario in genital CL is a construction worker who sleeps outdoors without full coverage. The glans and shaft of the penis are more likely to get involved. It initially presents as a small papule that gradually enlarges and becomes a crusty, ulcerated nodule with or without discharge. Regional lymphadenopathy may be present [117, 118].

#### 9.6.2 Diagnosis and Differential Diagnosis

Typically, genital ulceration is caused by sexually transmitted infections (STIs), including HSV, syphilis, lymphogranuloma venereum, granuloma inguinale, and chancroid. Other bacterial and fungal infections, such as Haemophilus ducreyi, TB, and candida balanitis, can be responsible, too. Noninfectious causes such as Behcet syndrome, Wegner granulomatosis, psoriasis, sexual trauma, and neoplasia should be considered as other differential diagnoses [117, 118].

Diagnosis is based on clinical appearance, considering local epidemiological data and evidence of Leishman bodies in tissue samples. In cases where a biopsy is unacceptable, the Leishmania skin test and smear of discharges may also aid in diagnosis [117, 118].

While there have been no reports of sexually transmitted CL in humans, it might be transmitted sexually in the visceral form of the disease, based on a single reported human case and veterinary literature [117].

#### 9.6.3 Treatment

In cases of genital CL, treatment options include IM meglumine antimoniate for 20 days and oral miltefosine 150 mg daily for 28 days [117, 118].

## 10 Study Limitations

One of the limitations of our study is the unavailable data analysis due to heterogeneous diagnostic methods from clinical examinations to para‐clinical methods reported in the included studies.

## 11 Conclusion

Old World cutaneous leishmaniasis may be misdiagnosed due to its unusual clinical presentations. Therefore, knowledge of the atypical features of OWCL can help clinicians make a better and prompt diagnosis and treatment.

## Author Contributions

**Bahareh Abtahi‐Naeini:** conceptualization, supervision, writing – review and editing. **Seyed Naser Emadi:** conceptualization, writing – review and editing. **Zabihollah Shahmoradi:** conceptualization, writing – review and editing. **Mahsa Pourmahdi‐Boroujeni:** writing – original draft. **Ali Saffaei:** writing – original draft. **Fereshte Rastegarnasab:** writing – original draft, writing – review and editing, project administration.

## Ethics Statement

The study is ethically approved by the “Research Ethics Committees of School of Medicine, Isfahan University of Medical Sciences, Isfahan, Iran” and the approval ID is: “IR.MUI.MED.REC.1402.376”.

## Conflicts of Interest

The authors declare no conflicts of interest.

## Transparency Statement

The lead author Seyed Naser Emadi, Fereshte Rastegarnasab affirms that this manuscript is an honest, accurate, and transparent account of the study being reported; that no important aspects of the study have been omitted; and that any discrepancies from the study as planned (and, if relevant, registered) have been explained.

## Supporting information

Supplement‐1.

Supplement‐2.

Supplement‐3.

Supplement‐4.

Supplement‐5.

Supplement‐6.

Supplement‐7.

Supplement‐8.

Supplement‐9.

Supplement‐10.

Supplement‐11.

Supplement‐12.

Supplement‐13.

Supplement‐14.

Supplement‐15.

Supplement‐16.

Supplement‐17.

Supplement‐18.

Supplement‐19.

Supplement‐20.

## Data Availability

The data that support the findings of this study are available from the corresponding author upon reasonable request.
