# Supplementary material for: Atypical Manifestations of Old World Cutaneous Leishmaniasis: A Systematic Review and Clinical Atlas of Unusual Clinical and Specific Anatomical Presentations
Source: Health Sci Rep. 2025 Sep 18;8(9):e71273. doi: 10.1002/hsr2.71273 (PMC12446576; doi:10.1002/hsr2.71273)
Supplement: Supplementary file 2 — Supplement‐2. [file HSR2-8-e71273-s011.docx]

**Supplement-2** Risk of Bias Assessment for the Included Studies using JBI Standard Critical Appraisal Tools. A) Case Reports, B) Case Series, and C) Analytical Cross-sectional Studies.

A)

| **Authors** | **Country** | **Year** | **Q1** | **Q2** | **Q3** | **Q4** | **Q5** | **Q6** | **Q7** | **Q8** |
| --- | --- | --- | --- | --- | --- | --- | --- | --- | --- | --- |
| Reinecke et al. | Germany | 2001 | Y | Y | Y | Y | N | Y | Y | Y |
| Al-Qattan | Saudi Arabia | 2002 | Y | N | Y | Y | Y | Y | Y | Y |
| Guarneri et al. | Italy | 2002 | Y | Y | Y | Y | Y | N | N | Y |
| Lahiry | Saudi Arabia | 2002 | N | N | Y | Y | Y | Y | Y | N |
| Shelleh et al. | Saudi Arabia | 2002 | Y | Y | Y | Y | Y | Y | Y | Y |
| Iftikhar et al. | Pakistan | 2003 | Y | N | Y | Y | Y | Y | Y | Y |
| Karincaoglu et al. | Turkey | 2004 | Y | N | Y | Y | Y | Y | N | N |
| Mencía-Gutiérrez et al. | Spain | 2005 | Y | Y | Y | Y | Y | Y | Y | Y |
| Sadeghian et al. | Iran | 2005 | Y | Y | Y | Y | Y | Y | Y | Y |
| Arfan et al. | Pakistan | 2006 | N | Y | Y | Y | Y | Y | N | Y |
| Manzur et al. | Pakistan | 2006 | N | Y | Y | Y | Y | Y | N | Y |
| Omidian et al. | Iran | 2006 | N | Y | Y | Y | Y | Y | Y | Y |
| Ejaz et al. | Pakistan | 2007 | Y | N | Y | Y | Y | Y | Y | Y |
| Esfandiarpour et al. | Iran | 2007 | Y | Y | Y | Y | Y | Y | Y | Y |
| Mahmood | Pakistan | 2007 | Y | Y | Y | Y | Y | Y | N | Y |
| Niamba et al. | Burkina Faso | 2007 | Y | Y | Y | Y | Y | Y | Y | Y |
| Ceyhan et al. | Turkey | 2008 | N | Y | Y | Y | Y | Y | Y | Y |
| Chaudhary et al. | India | 2008 | Y | Y | Y | Y | Y | N | N | Y |
| Nilforoushzadeh et al. | Iran | 2008 | Y | Y | Y | Y | Y | Y | Y | Y |
| Stefanidou et al. | Greece | 2008 | N | Y | Y | Y | Y | Y | Y | Y |
| Ceyhan et al. | Turkey | 2009 | N | Y | Y | Y | Y | N | Y | Y |
| Khorsandi-Ashtiani et al. | Iran | 2009 | Y | Y | Y | Y | Y | Y | Y | Y |
| Mehta et al. | India | 2009 | Y | Y | Y | Y | Y | N | N | Y |
| Eryilmaz et al. | Turkey | 2010 | N | Y | Y | Y | Y | Y | Y | Y |
| Yusuf et al. | Nigeria | 2010 | N | Y | Y | Y | Y | Y | Y | Y |
| Khaled et al. | Tunisia | 2011 | Y | Y | Y | Y | Y | Y | Y | Y |
| Mnejja et al. | Tunisia | 2011 | Y | Y | Y | Y | Y | N | N | Y |
| Poeppl et al. | Austria | 2011 | Y | Y | Y | Y | Y | Y | Y | Y |
| Robati et al. | Iran | 2011 | Y | Y | Y | Y | Y | Y | N | Y |
| Vasudevan et al. | India | 2011 | Y | Y | Y | Y | Y | Y | Y | Y |
| Masood et al. | Pakistan | 2012 | Y | Y | Y | Y | Y | Y | Y | Y |
| Purohit et al. | India | 2012 | Y | Y | Y | Y | Y | Y | N | Y |
| Verma et al. | India | 2012 | Y | Y | Y | Y | Y | Y | Y | Y |
| Alhumidi | Saudi Arabia | 2013 | N | Y | Y | Y | Y | N | N | Y |
| Ayatollahi et al. | Iran | 2013 | Y | Y | Y | Y | Y | Y | Y | Y |
| Yaich et al. | Tunisia | 2013 | Y | Y | Y | Y | Y | Y | Y | Y |
| Yesilova et al. | Turkey | 2013 | N | N | Y | Y | Y | Y | Y | Y |
| Ayatollahi et al. | Iran | 2014 | Y | Y | Y | Y | Y | Y | Y | Y |
| Ramot et al. | Israel | 2014 | Y | Y | Y | Y | N | N | N | Y |
| Siah et al. | UK | 2014 | Y | Y | Y | Y | Y | Y | Y | Y |
| Bandyopadhyay et al. | India | 2015 | Y | Y | Y | Y | N | Y | N | Y |
| Chiheb et al. | Morocco | 2015 | Y | Y | Y | Y | N | N | N | Y |
| Duman et al. | Turkey | 2015 | Y | Y | Y | Y | Y | Y | Y | Y |
| Ekiz et al. | Turkey | 2015 | Y | Y | Y | Y | Y | Y | N | Y |
| Salman et al. | Turkey | 2015 | Y | Y | Y | Y | Y | Y | N | Y |
| Bainz et al. | India | 2016 | Y | Y | Y | Y | N | Y | N | Y |
| Zhang et al. | China | 2016 | Y | Y | Y | Y | Y | Y | N | Y |
| Doroodgar et al. | Iran | 2017 | Y | Y | Y | Y | N | N | N | Y |
| Ozlu et al. | Turkey | 2017 | Y | Y | Y | Y | N | Y | N | Y |
| Patel | India | 2017 | Y | Y | Y | Y | Y | Y | N | Y |
| Saki et al. | Iran | 2017 | Y | Y | Y | Y | Y | Y | N | Y |
| Al-Dwibe et al. | Palestine | 2018 | Y | Y | Y | Y | Y | Y | N | Y |
| Badirzadeh et al. | Iran | 2018 | Y | Y | Y | Y | Y | Y | Y | Y |
| Hashemi et al. | Iran | 2018 | Y | Y | Y | Y | Y | Y | Y | Y |
| Kumari et al. | India | 2018 | Y | Y | Y | Y | Y | N | N | Y |
| Youssef et al. | Syria | 2018 | Y | Y | Y | Y | Y | Y | N | Y |
| Mosayebi et al. | Iran | 2019 | Y | Y | Y | Y | Y | Y | Y | Y |
| Rather et al. | India | 2019 | Y | Y | Y | Y | Y | Y | N | Y |
| Ahmad et al. | Syria | 2020 | Y | Y | Y | Y | Y | Y | Y | Y |
| Nabli et al. | Tunisia | 2020 | Y | Y | Y | Y | Y | Y | N | Y |
| Tegegne et al. | Ethiopia | 2020 | Y | Y | Y | Y | Y | Y | N | Y |
| Turkoglu et al. | Turkey | 2020 | Y | Y | Y | Y | Y | N | N | Y |
| Fellah et al. | Morocco | 2021 | Y | Y | Y | Y | Y | Y | Y | Y |
| Mahdavi et al. | Iran | 2021 | Y | Y | Y | Y | Y | Y | Y | Y |
| Nepal | Nepal | 2021 | Y | Y | Y | Y | Y | Y | Y | Y |
| Rodrigues et al. | India | 2021 | Y | N | Y | Y | Y | Y | N | Y |
| Bassaid et al. | Algeria | 2022 | Y | Y | Y | Y | Y | Y | N | Y |
| Chahboun et al. | Morocco | 2022 | Y | Y | Y | N | N | Y | N | Y |
| Diociaiuti et al. | Italy | 2022 | Y | Y | Y | Y | Y | Y | N | Y |
| Fakhar et al. | Iran | 2022 | Y | Y | Y | Y | Y | Y | N | Y |
| Gazerani et al. | Iran | 2022 | Y | Y | Y | Y | Y | Y | N | Y |
| Rekik et al. | Tunisia | 2022 | Y | Y | Y | Y | Y | Y | N | Y |
| Zhuang et al. | China | 2022 | Y | Y | Y | Y | Y | Y | N | Y |

B)

| **Authors** | **Country** | **Year** | **Q1** | **Q2** | **Q3** | **Q4** | **Q5** | **Q6** | **Q7** | **Q8** | **Q9** | **Q10** |
| --- | --- | --- | --- | --- | --- | --- | --- | --- | --- | --- | --- | --- |
| Dabiri et al. | Iran | 2001 | Y | Y | Y | N | N | Y | Y | N | Y | N |
| Bari et al. | Pakistan | 2009 | Y | Y | Y | N | N | Y | Y | Y | Y | N |
| Yaghoobi et al. | Iran | 2010 | Y | Y | Y | Y | Y | Y | Y | Y | Y | N |
| Sindhu et al. | India | 2012 | N | N | Y | N | N | Y | Y | N | Y | N |
| Dassoni et al. | Italy | 2013 | Y | Y | Y | N | N | Y | Y | Y | Y | N |
| Hajjaran et al. | Iran | 2013 | Y | Y | Y | N | N | Y | Y | N | Y | N |
| Moravvej et al. | Iran | 2013 | N | N | Y | N | N | Y | Y | Y | Y | N |
| Blasco et al. | Spain | 2014 | Y | Y | Y | N | N | Y | N | N | N | N |
| Talat et al. | Pakistan | 2014 | Y | Y | Y | N | N | Y | Y | Y | Y | N |
| Hayani et al. | Syria | 2015 | Y | Y | Y | N | N | Y | N | N | N | N |
| Thomaidou et al. | Israel | 2015 | Y | Y | Y | Y | Y | Y | N | N | Y | N |
| Mohammadpour et al. | Iran | 2016 | Y | Y | Y | Y | Y | Y | N | N | Y | N |
| Solomon et al. | Israel | 2016 | Y | Y | Y | Y | Y | Y | Y | N | Y | Y |
| Dassoni et al. | Ethiopia | 2017 | Y | Y | Y | Y | Y | N | Y | N | Y | N |
| Fernandez-Flores et al. | Spain | 2017 | Y | Y | Y | Y | Y | Y | N | N | Y | N |
| Gupta et al. | India | 2017 | Y | Y | Y | N | N | Y | Y | N | N | N |
| Kouki et al. | Tunisia | 2022 | Y | Y | Y | Y | Y | Y | Y | Y | Y | Y |

C)

| **Authors** | **Country** | **Year** | **Q1** | **Q2** | **Q3** | **Q4** | **Q5** | **Q6** | **Q7** | **Q8** |
| --- | --- | --- | --- | --- | --- | --- | --- | --- | --- | --- |
| Uzun et al. | Turkey | 2004 | Y | Y | Y | Y | NA | NA | Y | Y |
| Bari et al. | Pakistan | 2008 | Y | Y | Y | Y | NA | NA | Y | Y |
| Padovese et al. | Italy | 2009 | Y | Y | Y | Y | NA | NA | Y | Y |
| Sharifi et al. | Iran | 2010 | Y | Y | Y | Y | NA | NA | Y | Y |
| Douba et al. | Syria | 2012 | Y | Y | Y | Y | NA | NA | Y | Y |
| Saab et al. | Lebanon | 2012 | Y | Y | Y | Y | NA | NA | Y | Y |
| Khan et al. | Pakistan | 2016 | Y | Y | NA | Y | NA | NA | Y | Y |
| Khatri et al. | Italy | 2016 | Y | Y | NA | Y | NA | NA | Y | N |
| Siriwardana et al. | Sri Lanka | 2019 | Y | Y | NA | Y | NA | NA | Y | N |
| An et al. | Turkey | 2020 | Y | Y | NA | Y | NA | NA | Y | Y |
| Khan et al. | Pakistan | 2021 | Y | Y | NA | N | NA | NA | N | Y |
| Muthanna et al. | UK | 2022 | Y | Y | NA | Y | NA | NA | Y | N |

UK= United Kingdom, Y= Yes, N= No, NA= Not Applicable
