# Supplementary material for: Atypical Manifestations of Old World Cutaneous Leishmaniasis: A Systematic Review and Clinical Atlas of Unusual Clinical and Specific Anatomical Presentations
Source: Health Sci Rep. 2025 Sep 18;8(9):e71273. doi: 10.1002/hsr2.71273 (PMC12446576; doi:10.1002/hsr2.71273)
Supplement: Supplementary file 7 — Supplement‐7. [file HSR2-8-e71273-s015.docx]

**
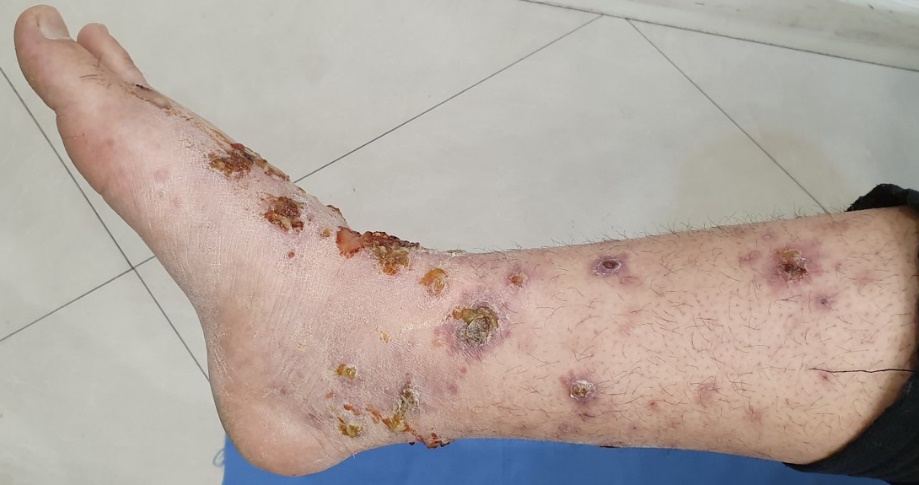
**

**Supplement-7** Eczematoid Cutaneous Leishmaniasis. Multiple ulcerated, purulent, eczematous lesions on the lower extremities (Photograph taken by Dr. Zabihollah Shahmoradi, Skin Diseases and Leishmaniasis Research Center, Isfahan University of Medical Sciences, Isfahan, Iran)
