# Supplementary material for: Atypical Manifestations of Old World Cutaneous Leishmaniasis: A Systematic Review and Clinical Atlas of Unusual Clinical and Specific Anatomical Presentations
Source: Health Sci Rep. 2025 Sep 18;8(9):e71273. doi: 10.1002/hsr2.71273 (PMC12446576; doi:10.1002/hsr2.71273)
Supplement: Supplementary file 10 — Supplement‐10. [file HSR2-8-e71273-s017.docx]

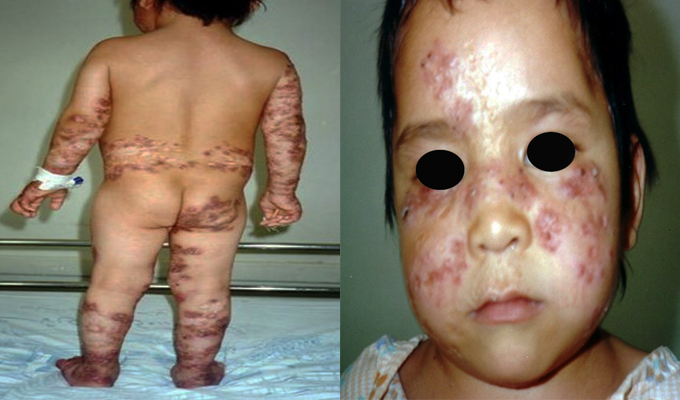


**Supplement-10** Diffuse Cutaneous Leishmaniasis. An infant covered with multiple erythematous, non-ulcerated papules and plaques. (Photograph taken by Dr. Zabihollah Shahmoradi, Skin Diseases and Leishmaniasis Research Center, Isfahan University of Medical Sciences, Isfahan, Iran)
