# Supplementary material for: Atypical Manifestations of Old World Cutaneous Leishmaniasis: A Systematic Review and Clinical Atlas of Unusual Clinical and Specific Anatomical Presentations
Source: Health Sci Rep. 2025 Sep 18;8(9):e71273. doi: 10.1002/hsr2.71273 (PMC12446576; doi:10.1002/hsr2.71273)
Supplement: Supplementary file 11 — Supplement‐11. [file HSR2-8-e71273-s004.docx]

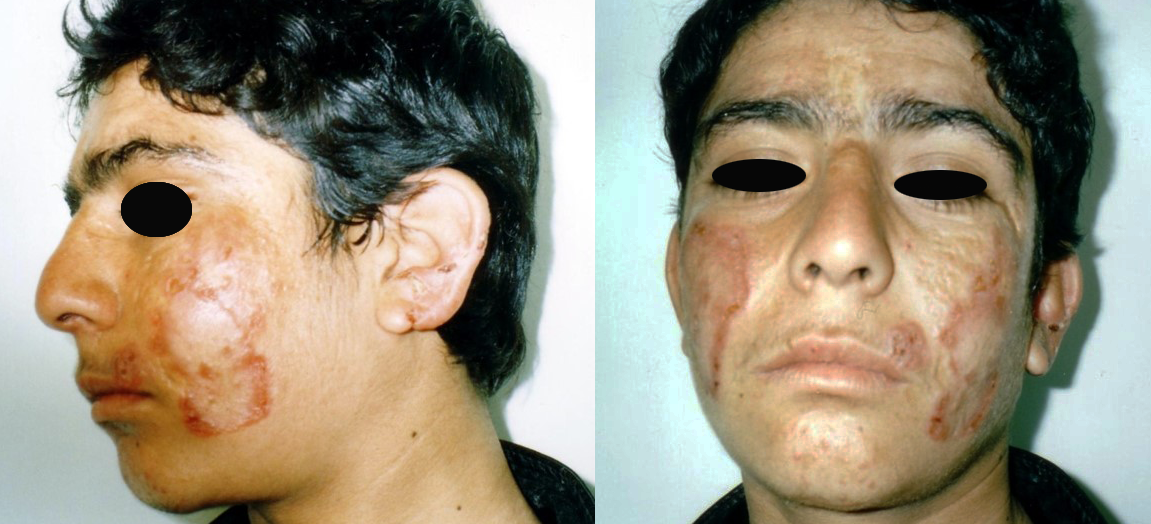


**Supplement-11** Leishmaniasis Recidivans. A man with erythematous, yellow-reddish facial papules and plaques, located over the scar of previously healed CL lesions. (Photograph taken by Dr. Zabihollah Shahmoradi, Skin Diseases and Leishmaniasis Research Center, Isfahan University of Medical Sciences, Isfahan, Iran)
