# Supplementary material for: Atypical Manifestations of Old World Cutaneous Leishmaniasis: A Systematic Review and Clinical Atlas of Unusual Clinical and Specific Anatomical Presentations
Source: Health Sci Rep. 2025 Sep 18;8(9):e71273. doi: 10.1002/hsr2.71273 (PMC12446576; doi:10.1002/hsr2.71273)
Supplement: Supplementary file 15 — Supplement‐15. [file HSR2-8-e71273-s013.docx]

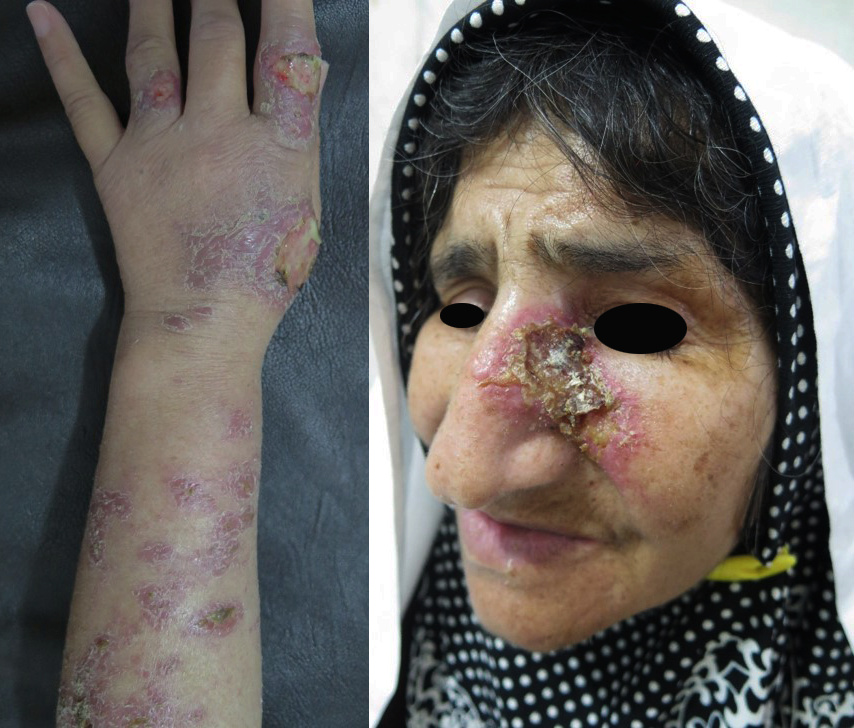


**Supplement-15** Co-existence with Cancer Chemotherapy or Immunosuppressive Treatment. Multiple erythematous, crusted ulcers on the face and upper limb of a woman involved with breast cancer. (Photograph taken by Dr. Zabihollah Shahmoradi, Skin Diseases and Leishmaniasis Research Center, Isfahan University of Medical Sciences, Isfahan, Iran)
