# Supplementary material for: Atypical Manifestations of Old World Cutaneous Leishmaniasis: A Systematic Review and Clinical Atlas of Unusual Clinical and Specific Anatomical Presentations
Source: Health Sci Rep. 2025 Sep 18;8(9):e71273. doi: 10.1002/hsr2.71273 (PMC12446576; doi:10.1002/hsr2.71273)
Supplement: Supplementary file 18 — Supplement‐18. [file HSR2-8-e71273-s012.docx]

**
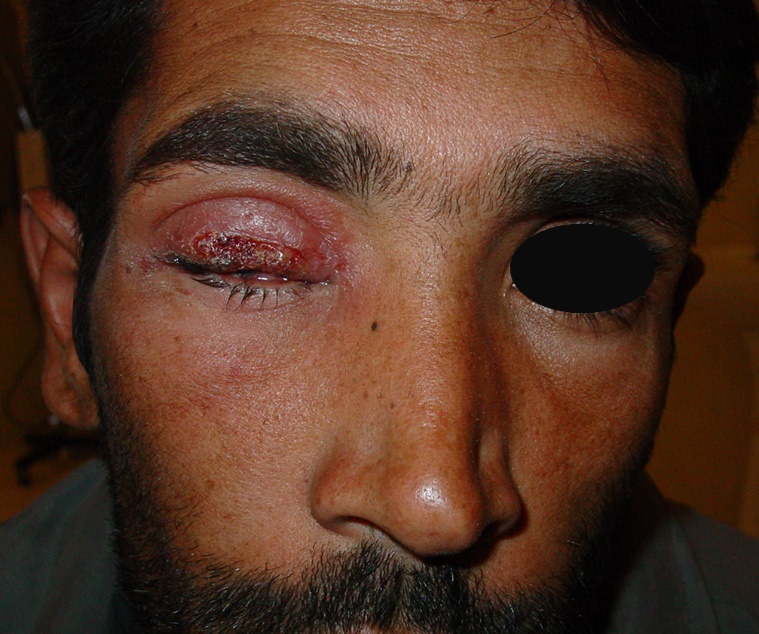
**

**Supplement-18** Ocular Cutaneous Leishmaniasis. An erythematous, indurated lesion on the upper eyelid leading to ptosis. (Photograph taken by Dr. Seyed Naser Emadi, Skin Research Center of Razi and Imam Khomeini Hospital and Research Center for War-affected People, Tehran University of Medical Sciences, Tehran, Iran)
