# Supplementary material for: Atypical Manifestations of Old World Cutaneous Leishmaniasis: A Systematic Review and Clinical Atlas of Unusual Clinical and Specific Anatomical Presentations
Source: Health Sci Rep. 2025 Sep 18;8(9):e71273. doi: 10.1002/hsr2.71273 (PMC12446576; doi:10.1002/hsr2.71273)
Supplement: Supplementary file 19 — Supplement‐19. [file HSR2-8-e71273-s008.docx]

**
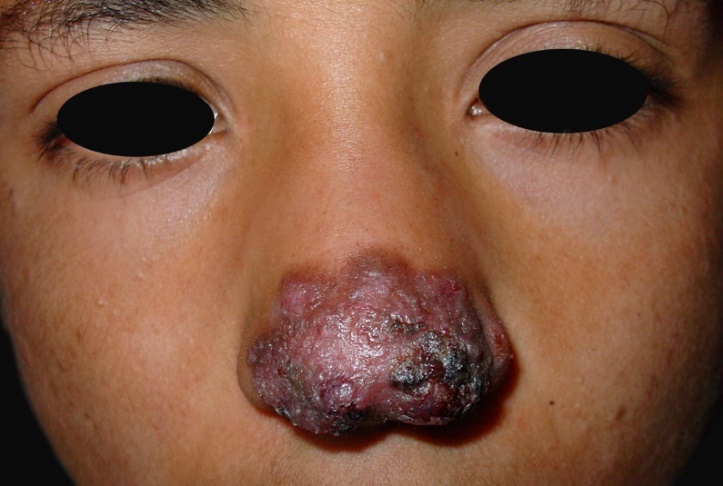
**

**Supplement-19** Nasal Cutaneous Leishmaniasis. A large rhinophyma-like satellite lesion. (Photograph taken by Dr. Seyed Naser Emadi, Skin Research Center of Razi and Imam Khomeini Hospital and Research Center for War-affected People, Tehran University of Medical Sciences, Tehran, Iran)
